# LvCD14L Acts as a Novel Pattern Recognition Receptor and a Regulator of the Toll Signaling Pathway in Shrimp

**DOI:** 10.3390/ijms24097770

**Published:** 2023-04-24

**Authors:** Xinjia Lv, Shihao Li, Yang Yu, Songjun Jin, Xiaojun Zhang, Fuhua Li

**Affiliations:** 1 CAS and Shandong Province Key Laboratory of Experimental Marine Biology, Institute of Oceanology, Chinese Academy of Sciences, Qingdao 266071, China; 2 Laboratory for Marine Biology and Biotechnology, Qingdao National Laboratory for Marine Science and Technology, Qingdao 266237, China; 3 Center for Ocean Mega-Science, Chinese Academy of Sciences, Qingdao 266071, China; 4 The Innovation of Seed Design, Chinese Academy of Sciences, Wuhan 430072, China

**Keywords:** pattern recognition receptor, CD14, toll, NF-κB pathway, *Vibrio parahaemolyticus*, *Litopenaeus vannamei*

## Abstract

Leucine-rich repeat (LRR) is a structural motif has important recognition function in immune receptors, such as Tolls and NOD-like receptors (NLRs). The immune-related LRR proteins can be divided into two categories, LRR-containing proteins and LRR-only proteins. The latter contain LRR motifs while they are without other functional domains. However, the functional mechanisms of the LRR-only proteins were still unclear in invertebrates. Here, we identified a gene encoding a secretory LRR-only protein, which possessed similarity with vertebrate CD14 and was designated as *LvCD14L*, from the Pacific whiteleg shrimp *Litopenaeus vannamei*. Its transcripts in shrimp hemocytes were apparently responsive to the infection of *Vibrio parahaemolyticus*. Knockdown of *LvCD14L* with dsRNA resulted in significant increase of the viable bacteria in the hepatopancreas of shrimp upon *V. parahaemolyticus* infection. Further functional studies revealed that LvCD14L could bind to microorganisms’ PAMPs, showed interaction with LvToll1 and LvToll2, and regulated the expression of *LvDorsal* and *LvALF2* in hemocytes. These results suggest that LvCD14L functions as a pattern recognition receptor and activates the NF-κB pathway through interaction with LvTolls. The present study reveals a shrimp LvCD14L-Tolls-NF-κB signaling pathway like the CD14/TLR4/NF-κB signaling pathway in mammalians, which enriches the functional mechanism of secretory LRR-only immune receptors during pathogens infection in invertebrates.

## 1. Introduction

Shrimp is an important aquaculture species in China. In recent years, bacterial diseases have seriously affected the healthy development of the shrimp aquaculture industry. As an invertebrate, shrimp mainly relies on innate immunity to resist pathogen infections. Recognition of pathogens is an important step of the immune response of hosts. In innate immunity, the host initiates an immune response by pattern recognition receptors (PRRs) recognizing pathogen-associated molecular patterns (PAMPs). Therefore, identification of novel PRRs in shrimp is crucial for understanding the host immune response.

Leucine-rich repeat (LRR) is a structural motif which widely exists in thousands of proteins from viruses to eukaryotes [1,2,3]. An LRR motif usually consists of 20 to 30 residues forming a β strand-α helix structure [4]. LRR proteins, containing two or more LRR motifs, provide a curved solenoid structural framework for protein–protein interaction, and their concave surfaces usually contain ligand-binding sites [4]. In addition to LRR motifs, N-terminal (LRRNT) and C-terminal (LRRCT) capping motifs are common motifs in extracellular and membrane-associated LRR proteins for protecting the hydrophobic inner core of the solenoid [5,6]. Both capping motifs usually contain four cysteines, which form disulfide bonds for stabilizing the structure. LRR proteins are involved in many physiological processes, including cell adhesion, extracellular matrix assembly, neuronal development, RNA processing, and immune responses [7].

The immune-related LRR proteins have important functions as immune receptors and could be mainly divided into two categories: the one includes proteins with LRR motifs and other functional domains, such as Toll-like receptors (TLRs) and NOD-like receptors (NLRs) [8,9]; another one contains LRR-only proteins, such as variable lymphocyte receptors (VLRs) and cluster of differentiation 14 (CD14) [10,11]. These molecules usually act as pattern recognition receptors (PRRs), which play important functions in the innate immunity of animal kingdoms. TLRs and NLRs widely exist in metazoan animals, while VLRs are mainly reported in jawless vertebrates and CD14s are only reported in high vertebrate animals [12,13].

In addition to the classical immune receptors, some other LRR-containing proteins are also important in immune responses in invertebrates. In *Marsupenaeus japonicus*, the soluble PRR, Leulectin, comprising LRR motifs and a C-type lectin-like domain (CTLD), could recognize flagellin through the LRRs and agglutinate the bacteria and promote hemocytic phagocytosis by CTLD [14]. Some LRR-only proteins were also identified serving as important immune receptors in invertebrates. In *Bathymodioline mussels*, BpLRR-1 could recognize LPS and serve as an intracellular recognition receptor for endosymbionts [15]. In *Chlamys farreri*, the LRR-only protein CfLRRop-1 could bind to different PAMPs and induce the release of TNF-alpha in hemocytes of scallop [16]. In the sea cucumber *Apostichopus japonicus*, the LRR-only protein Aj-VLRA, which shared a similar structure and function with the type A VLR protein, was responsive to pathogen challenge and bound to both Gram-positive and Gram-negative bacteria [17]. The immune functions of LRR-only proteins were also reported in shrimp and crab. A transmembrane LRR-only protein, LvLRRm, identified from the shrimp *Litopenaeus vannamei* played important roles in shrimp against *Vibrio* infection [18]. Two LRR-only proteins, PtLRR1 and PtLRR2, identified from the swimming crab *Portunus trituberculatus* displayed different regulatory activities on the expression of proPO system and inflammation-related genes [19]. However, the underlaying mechanisms of how LRR-only proteins function in host immune system are still largely unknown.

In the present study, we identified a gene, designated as *LvCD14L*, encoding LRR-only protein from the shrimp *L. vannamei*. *LvCD14L* played an important role during *Vibrio parahaemolyticus* infection. Functional studies revealed that LvCD14L acted as a pattern recognition receptor (PRR), which could bind to shrimp Tolls and activate the NF-κB pathway. The present study provides new evidence for clarifying the functional mechanisms of LRR-only receptors in invertebrates during pathogens infection.

## 2. Results

### 2.1. LvCD14L Encodes a Secretory LRR-Only PROTEIN

**Figure 1:**
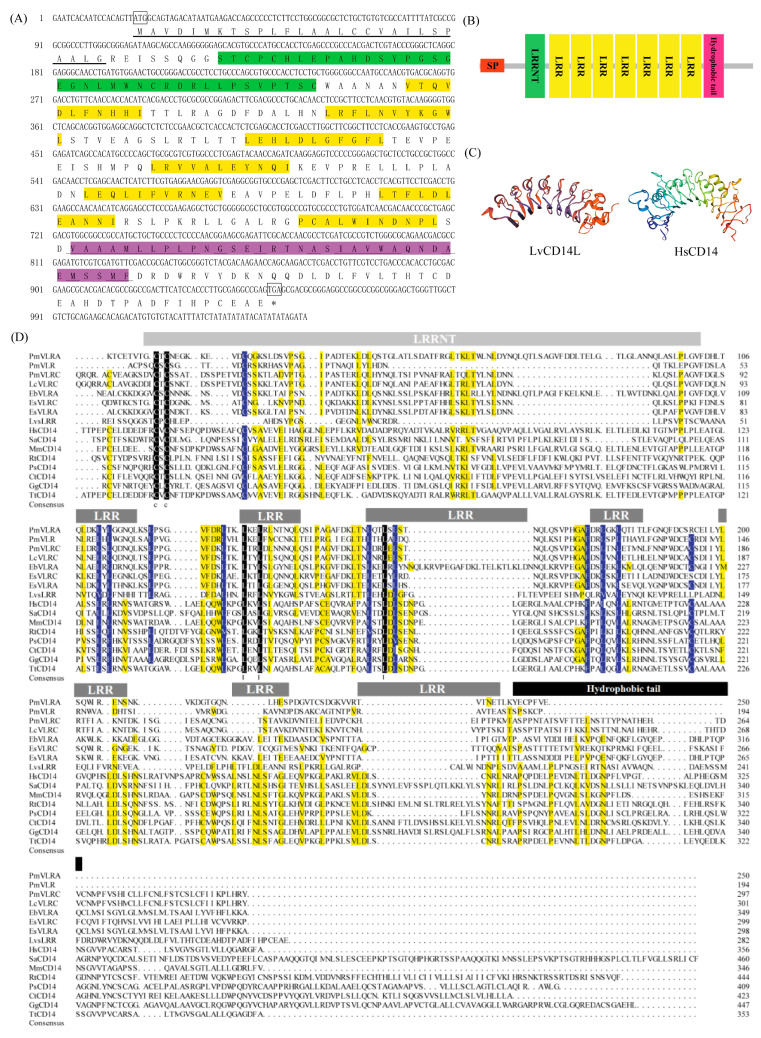
Sequence features of LvCD14L. (**A**) Nucleotide and amino acid sequences of LvCD14L. The start codon and stop codon were boxed. The predicted signal peptide was underlined. The LRRNT, LRR and hydrophobic tail were marked with green, yellow and pink background. The hydrophobic amino acid residues in hydrophobic tail were marked with a wavy line. (**B**) The domain composition of LvCD14L. SP, signal peptide; LRRNT, N-terminal LRR domain; LRR, Leucine-rich repeat; Hydrophobic tail, hydrophobic amino acid-rich region. (**C**) Multiple sequences alignment of VLR genes and CD14 genes from different species. Identical and similar residues were marked with black, blue and yellow, respectively. Sequence information of VLR and CD14 genes is shown in Table S3. (**D**) The three-dimensional (3D) structure modeling of LvCD14L. The model of LvCD14L adopted a horseshoe-shaped structure, which was similar to other LRR proteins.

The open reading frame of *LvCD14L* had 918 bp nucleotides, encoding a protein of 310 amino acid residues (Figure 1A). The amino acid sequence of LvCD14L contained a 28 aa signal peptide, a LRRNT region (Ser^32^-Cys^69^), seven LRR regions (Val^76^-Ile^86^, Leu^100^-Leu^110^, Leu^124^-Leu^134^, Leu^147^-Ile^157^, Leu^172^-Val^182^, Leu^194^-Ile^204^ and Pro^218^-Leu^228^) and a hydrophobic tail (Val^231^-Phe^265^), while it lacked the LRRCT region of LRR protein, which was like CD14 from vertebrates (Figure 1B). In the hydrophobic tail, the hydrophobic amino acid residues account for 60% of the total amino acid residues. Sequence alignment analysis showed that the amino acid sequence of LvCD14L had a similarity ranging from 19.44% to 24.12% with CD14s, and had a similarity ranging from 26.32% to 33.85% with VLRs from jawless vertebrates (Figure 1D). The three-dimensional structure showed that LvCD14L adopted a horseshoe-shaped structure, which was similar to other LRR proteins (Figure 1C).

**Figure 2:**
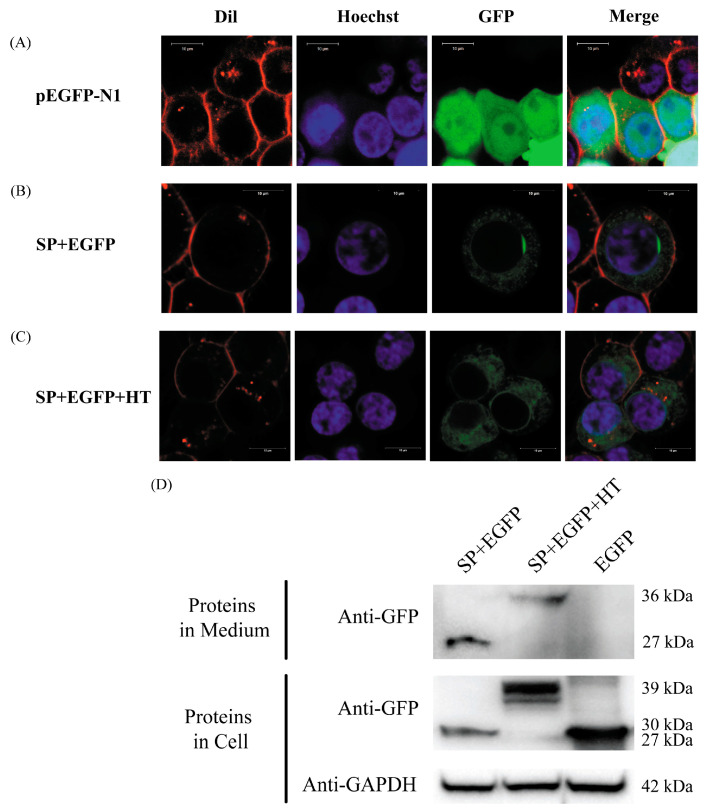
Cellular localization of LvCD14L in 293T cells. Nucleus was visualized with Hoechst. Cell membrane was dyed by Dil. (**A**) 293T cells transfected with pEGFP-N1 plasmid. (**B**) 293T cells transfected with SP + EGFP plasmid. (**C**) 293T cells transfected with SP + EGFP + HT (hydrophobic tail) plasmid. The minor band is the non-specific binding of anti GFP-Tag mAb to other proteins in 293T cells. (**D**) Detection of recombinant proteins in cell culture medium and cell by Western blot.

The subcellular localization analysis showed that LvCD14L was predicted as a cell membrane or extracellular protein. The cellular localization experiment further confirmed that LvCD14L was an extracellular protein. The recombinant protein consisting of LvCD14L signal peptide (SP), EGFP and LvCD14L hydrophobic tail (HT) had a weak signal in cytoplasm (Figure 2C) compared with the signal of EGFP (Figure 2A), which was similar to the recombinant protein consisting of SP and EGFP (Figure 2B). No positive signal was detected on the cell membrane when transfected with the SP + EGFP + HT plasmid. The result of the Western blot showed that the recombinant protein of SP + EGFP and SP + EGFP + HT could be detected in the cell lysate and medium, while the EGFP was detected in the cell lysate (Figure 2D).

### 2.2. LvCD14L Participates in V. parahaemolyticus Infection

**Figure 3:**
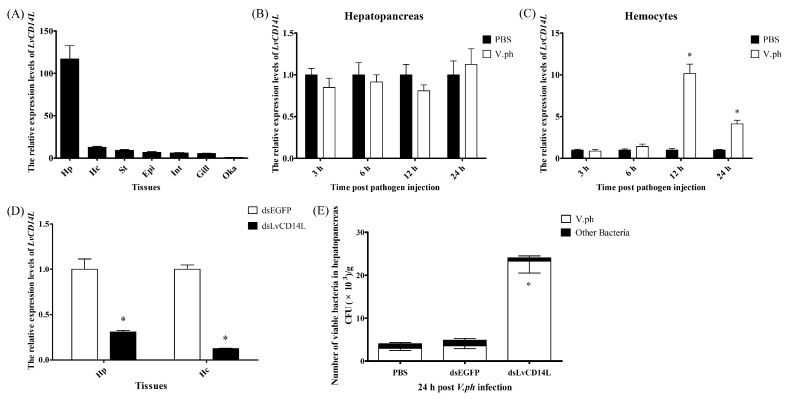
Expression patterns and immune function of *LvCD14L* during *V. parahaemolyticus* infection. (**A**) Tissue distribution of *LvCD14L* transcripts. Vertical bars represented mean ± S.E (*n* = 9). Epi, epidermis; Int, intestine; Hp, hepatopancreas; Gi, gill; St, stomach; Hc, hemocytes; Oka, lymphoid organ. Time-course expression pattern of LvCD14L after *V. parahaemolyticus* challenge in hepatopancreas (**B**) and hemocytes (**C**). Significant differences between treatment and control groups were labeled with an asterisk at *p* < 0.05. (**D**) Silencing efficiency of *LvCD14L* dsRNA in hepatopancreas and hemocytes. “dsLvCD14L” and “dsEGFP” indicate the group injected with LvCD14L dsRNA and EFGP dsRNA. The effective silencing dose is marked with an asterisk at *p* < 0.05 (*n* = 9). (**E**) The total viable bacteria counts in the hepatopancreas of LvCD14L-silenced shrimp after *V. parahaemolyticus* injection. The “V.ph” indicates the amount of *V. parahaemolyticus* in shrimp from different treatments. The “other bacteria” indicates the amount of bacteria, including *Vibrio harveyi* and *Vibrio brasiliensis*. The data were obtained from three independent repeats. Significant differences between the amount of *V. parahaemolyticus* in shrimps of treatment and control groups are labeled with an asterisk at *p* < 0.05 (*n* = 9).

Tissue distribution analysis showed that *LvCD14L* was widely expressed in all detected tissues, with the highest expression level in the hepatopancreas, followed by the hemocytes, stomach, epidermis, intestine, gill and lymphoid organ (Oka) (Figure 3A). The time-course expression patterns of *LvCD14L* in the hepatopancreas and hemocytes of shrimp after *V. parahaemolyticus* challenge were analyzed. In the hepatopancreas, the expression level of *LvCD14L* had no significant difference at all detected time points between the challenge group and the control group (Figure 3B). In hemocytes, the expression level of *LvCD14L* was up-regulated by 10.16-fold and 4.14-fold at 12 h and 24 h after *V. parahaemolyticus* challenge, respectively (Figure 3C).

To detect the immune function of *LvCD14L*, the influence of *LvCD14L* knockdown on *V. parahaemolyticus* propagation in shrimp was analyzed. *LvCD14L* was knocked down by dsRNA at the dosage of 6 μg per individual, with an interference efficiency of 69.09% and 87.52% in the hepatopancreas and hemocytes, respectively (Figure 3D). The number of bacteria in the hepatopancreas was an indicator for the health condition of shrimp. To study the impact of *LvCD14L* silencing on the *V. parahaemolyticus* infection process, the amount of *Vibrio* in the hepatopancreas of *LvCD14L*-silenced shrimp was detected. At 24 h after pathogen injection, the amount of *V. parahaemolyticus* in hepatopancreas of shrimp from the dsLvCD14L group was 2.33 × 10^4^ cfu/g, which was 6.59-fold higher than that from the dsEGFP group (3.53 × 10^3^ cfu/g) (Figure 3E). The number of other bacteria, including *V. harveyi* and *Vibrio brasiliensis*, did not show a significant difference between the dsLvCD14L group and control groups. The count of neither *V. parahaemolyticus* nor other bacteria had differences between the dsEGFP group and the PBS group. The results suggested that *LvCD14L* participated in *V. parahaemolyticus* infection.

### 2.3. LvCD14L Acts as a Pattern Recognition Receptor

**Figure 4:**
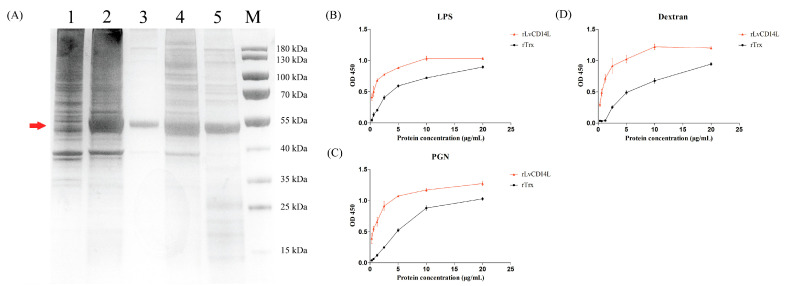
Binding activities of LvCD14L with different PAMPs. (**A**) SDS-PAGE of rLvCD14L produced in *E. coli* expression system. The expected band of rLvCD14L is indicated by an arrow. Lane 1: total protein of *E. coli* before induction; Lane 2: total protein of *E. coli* after induction; Lane 3: inclusion of the induced *E. coli* lysate; Lane 4: supernatant of the induced *E. coli* lysate; Lane 5: purified rLvCD14L; Lane M: Protein ladder marker. ELISA analysis of binding activity of rLvCD14L and rTrx to (**B**) lipopolysaccharide (LPS), (**C**) peptidoglycan (PGN) and (**D**) dextran. Results were obtained based on three independent repeats.

The recombinant LvCD14L protein (rLvCD14L) was expressed in *E. coli* after IPTG induction with a predicted molecular mass of 50.04 kDa (Figure 4A, lane 1 and 2). The induced rLvCD14L protein was expressed in both the inclusion body and soluble form (Figure 4A, lane 3 and 4) and was then purified (Figure 4A, lane 5). The results of ELISA showed that the rLvCD14L protein possessed concentration-dependent binding activities to PGN, LPS and dextran, respectively (Figure 4B–D). The MIC assay and agglutinating assay showed that rLvCD14L did not have antimicrobial activity (Table S1) or agglutinating activity (Figure S1). These results suggested that LvCD14L functioned as a PRR through direct binding to PAMPs.

### 2.4. LvCD14L Activates the TLR-Dorsal Pathway through Binding to LvTolls

**Figure 5:**
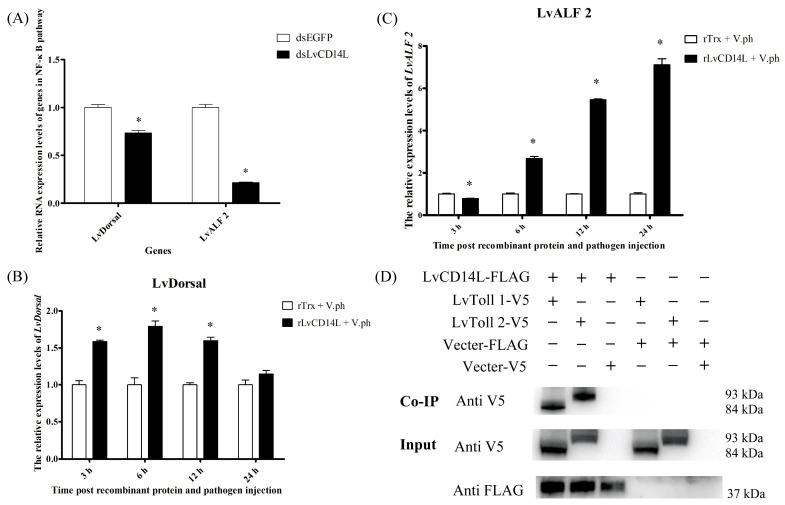
LvCD14L regulates Dorsal-mediated AMP expression through binding to LvTolls. Significant differences between treatment and control groups were labeled with an asterisk at *p* < 0.05. (**A**) The expression levels of genes in NF-κB pathways after LvCD14L knockdown. (**B**) Time-course expression pattern of *LvDorsal* in hemocytes after rLvCD14L and *V. parahaemolyticus* injection. (**C**) Time-course expression pattern of *LvALF2* in hemocytes after rLvCD14L and *V. parahaemolyticus* injection. (**D**) Results of co-immunoprecipitation. Sf9 cells were transfected with plasmids expressing LvCD14L-FLAG (FLAG-tagged LvCD14L), LvToll1-V5 (V5-tagged LvToll1), LvToll2-V5 (V5-tagged LvToll2) or empty plasmid (vector). The Co-IP results confirmed by Western blot using anti-V5 antibody as a probe. The input samples were detected by Western blot using anti-V5 and anti-FLAG antibody as a probe, respectively. Results were obtained based on three independent repeats.

As a PRR, LvCD14L might activate immune pathways when the host encounters pathogens infection. Therefore, we detected the expression changes of several immune-related transcription factors and antimicrobial peptide genes when *LvCD14L* was knocked down. The results showed that the NF-κB transcription factor *LvDorsal* was down-regulated in hemocytes after *LvCD14L* knockdown (Figure 5A). Simultaneously, the antimicrobial peptide gene *LvALF2* was also down-regulated in hemocytes (Figure 5A). Overexpression of LvCD14L further confirmed the regulation of LvCD14L on the NF-κB pathway. Injection of rLvCD14L significantly up-regulated the expression level of *LvDorsal* in hemocytes at 3 hpi, 6 hpi and 12 hpi compared with that in the rTrx injection group (Figure 5B). The expression level of *LvALF2* in hemocytes was up-regulated at 6 hpi, 12 hpi and 24 hpi (Figure 5C). These results suggested that LvCD14L could activate the NF-κB pathway in shrimp during *V. parahaemolyticus* infection.

As a secretory protein, LvCD14L needs a transmembrane protein to transduce the extracellular immune signal inside the cells. The Dorsal is the core transcription factor of the TLR-Dorsal signaling pathway. Therefore, we guessed that LvCD14L might bind to the extracellular part of LvTolls. As LvToll1 was reported to be responsive to *Vibrio* infection and has a close evolutionary relationship with LvToll2, we detected the interaction of LvCD14L with LvToll1 and LvToll2. The Co-IP assay showed that LvCD14L could interact with LvToll1 and LvToll2 (Figure 5D). The results suggested that LvCD14L could bind to LvTolls and activate the NF-κB pathway in shrimp upon pathogens infection.

## 3. Discussion

As immune receptors, LRR-only proteins mainly function through recognition of pathogens and activation of subsequent immune responses. In mammalians, CD14 was initially found to bind complexes of LPS-LBP and enhance host responses to LPS [20], then was found to bind PGN and LTA [21,22]. In jawless vertebrates, VLRs can not only specifically recognize protein antigens but also exhibit great specificity for glycan [23]. In invertebrates, some immune-related LRR-only proteins with pattern recognition function can also bind to microorganisms or PAMPs [16,17] as well as affect the expression of genes in the NF-κB pathway including Dorsal and AMPs [18]. In the present study, the LvCD14L protein could bind to different PAMPs, including LPS, PGN and dextran, and affect the expression of genes in the NF-κB pathway, suggesting that LvCD14L functions as a PRR, which recognizes pathogens’ PAMPs and activates host immune responses.

The most prominent biological functions of PRRs are recognition of PAMPs and initiation of signal transduction. The membrane-associated TLR recognizes PAMPs or ligands by its extracellular LRR domain and then transduces immune signals into the cells through its intracellular TIR domain interacting with an adaptor protein [24]. The cytoplastic NOD-like receptor (NLR), another LRR domain-containing PRR, activates subsequent immune responses using its N-terminal domain upon binding to PAMPs [25]. These kinds of PRRs have typical domains for signal transduction. Secretory PRRs also play critical roles in humoral immunity and can bind and eliminate microbes through multiple mechanisms including agglutination, neutralization, opsonization and complement activation [26,27,28]. In vertebrates, secretory PRRs could also interact with and regulate the function of membrane-associated PRRs such as soluble TLR2 (sTLR2) and sCD14 [29,30,31]. In invertebrates, some secretory LRR-containing proteins, such as CfLRRop-7 from *Chlamys farreri* and Leureptin from *Manduca sexta*, were regarded as PRRs because they could bind to PAMPs and induce antimicrobial peptides’ expression or hemocyte responses upon infection [32,33]. Vertebrate CD14 has two forms, with one anchored to the cell membrane by GPI and another one secreted into the extracellular space. The signal peptide (SP) and hydrophobic tail (HT) play crucial roles in the cellular localization of glycosylphosphatidylinositol (GPI)-anchored proteins. As LvCD14L has a predicted hydrophobic tail, three plasmids were constructed to test whether LvCD14L was cell membrane-anchored or secreted extracellularly. “EGFP-N1” was a control plasmid, which expressed EGFP intracellularly. “SP + EGFP” was jointly constructed with the nucleotide sequence encoding the signal peptide of LvCD14L and EGFP, which mainly expressed protein secreted extracellularly. As SP and HT should be added at the N-terminal and C-terminal of EGFP, respectively, the whole LvCD14L was not used to jointly construct the plasmid “SP + EGFP + HT”, which had a similar expression pattern at the protein level like “SP + EGFP”. The Western blot analysis further confirmed the result. In addition, no signal was detected on the cell membrane when transfected with these plasmids. Therefore, LvCD14L was a secretory protein and the predicted hydrophobic tail did not have the function of cell membrane anchoring.

**Figure 6:**
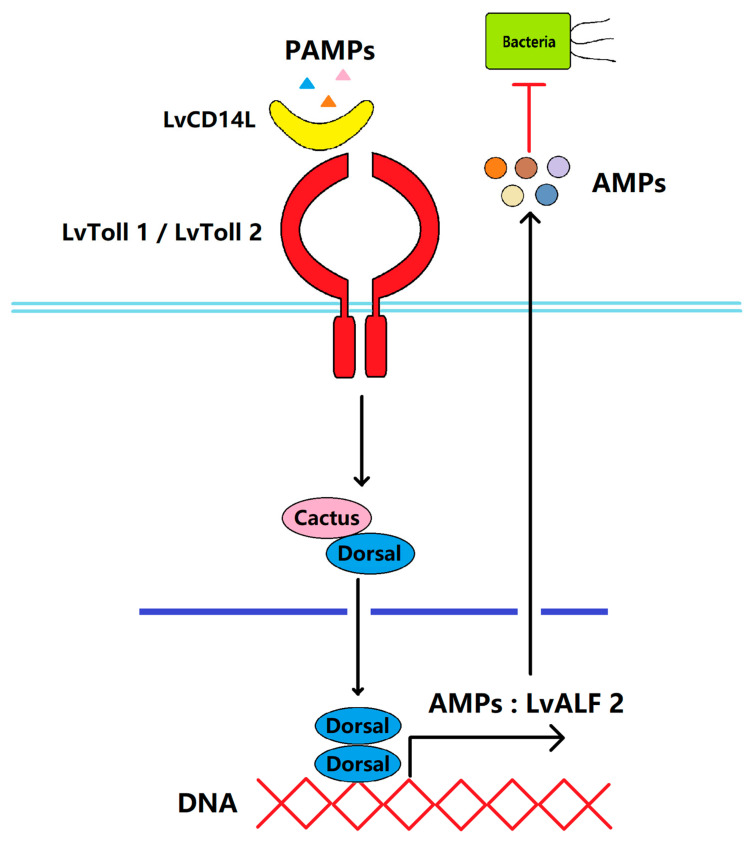
Model for LvCD14L-mediated antibacterial mechanism against *V. parahaemolyticus*. LvCD14L sensed *V. parahaemolyticus* and bound to LvToll1 or LvToll2 and activated NF-κB pathways. The transcription factor Dorsal translocated into the nucleus and led to the transcription of some AMPs such as *LvALF2*.

In the present study, we found that LvCD14L modulated the NF-κB pathway and AMP expression, which was consistent with previously reported extracellular LRR-containing PRRs. The NF-κB pathway is a classical immune signal transduction pathway that is mediated by the membrane-associated receptor Tolls and TLRs [34]. The extracellular region of Tolls and TLRs consist of multiple LRR motifs for PAMPs and ligands’ binding. Notably, LRR motifs could form homologous or heterogeneous polymers [35]. Therefore, we speculated that LvCD14L protein probably activates intracellular immune responses through interaction with shrimp Tolls. In *L. vannamei*, a total of eleven Toll genes have been identified and all of them are responsive to *V. parahaemolyticus* infection [36]. Particularly, functional studies further revealed the involvement of LvToll1 and LvToll2 during *Vibrio* infection [37,38]. Meanwhile, LvToll1 and LvToll2 could regulate the NF-κB-dependent antimicrobial peptides’ (AMPs) expression [39,40]. Therefore, LvToll1 and LvToll2 were considered as the target membrane-associated PRRs for LvCD14L interaction, which was proved by the protein–protein interaction analysis. Therefore, a working model was proposed to illustrate how LvCD14L functions during *V. parahaemolyticus* infection (Figure 6). After pathogen infection, LvCD14L protein functions as a PRR by recognizing PAMPs and then interacting with LvTolls and activating AMP production.

As the classical immune receptors in the innate immunity, Tolls or TLRs receive extracellular signals in different ways in invertebrates and mammalians. In *Drosophila*, Tolls cannot recognize PAMPs directly while they need the cytokine Spätzle [41]. Spätzle was activated by a series of protease cascades initiated from the recognition of PAMPs by molecules such as Gram-negative binding protein (GNBP) 3 and PGN recognition protein (PGRP)-SA [42]. TLR4, the first discovered mammalian homologue of *Drosophila* Toll, recognizes LPS delivered by LPS binding protein and CD14, and transduces the signal into the cells [9]. To date, despite several Spätzle-like genes being identified in shrimp, there was no evidence that showed these Spätzle-like proteins could interact with shrimp Tolls [43]. Tolls in shrimp can directly recognize PAMPs, which is different from that in *Drosophila* [34,44]. Therefore, researchers proposed that the Toll pathway in shrimp was similar to the mammalian TLR pathway [34]. In the present study, LvCD14L functions similar to the CD14 in mammalians, which supports the previous viewpoint. The present data suggest that there is a LvCD14L-Tolls-NF-κB signaling pathway similar to the CD14/TLR4/NF-κB signaling pathway in mammalians.

## 4. Materials and Methods

### 4.1. Animal and Tissues Collection

Healthy shrimp *L. vannamei*, with an average body weight of 5.36 g were cultured in an indoor breeding room and fed with an artificial diet. Different tissues including the intestine, stomach, gill, epidermis, hepatopancreas and lymphoid organ (Oka) were collected from nine individuals. Hemolymph was extracted using a syringe with an equal volume of anticoagulant (27 mM sodium citrate, 336 mM NaCl, 115 mM glucose, 9 mM EDTA, pH 7) and centrifuged at 800× *g*, 4 °C for 10 min to collect hemocytes. All the samples were preserved in liquid nitrogen for total RNA extraction and gene expression analysis.

### 4.2. Total RNA Extraction and cDNA Synthesis

Total RNA of different tissues was extracted with RNAiso Plus reagent (TaKaRa, Shiga, Japan), following the manufacturer’s protocol. The concentration of each RNA sample was measured by Nanodrop 2000 (Thermo Fisher Scientific, Waltham, MA, USA). The quality of RNA samples was assessed by electrophoresis on 1% agarose gel. The cDNA was synthesized from 1 μg total RNA using PrimeScript RT Reagent Kit (TaKaRa, Shiga, Japan), according to the manufacturer’s instruction. Briefly, the genomic DNA (gDNA) was removed with gDNA Eraser. Then, the first-strand cDNA was synthesized by PrimeScript RT Enzyme with random primers.

### 4.3. Gene Cloning

The cDNA sequence of *LvCD14L* was identified from a transcriptome database of *L. vannamei* [45]. Primers *LvCD14L*-F and *LvCD14L*-R (Table S2) were designed to amplify the *LvCD14L* gene from the cDNA sample of shrimp hepatopancreas. The PCR was performed using the Premix Ex Taq Hot Start version (TaKaRa, Shiga, Japan), and the program contained 35 cycles of 98 °C for 10 s, 60 °C for 30 s and 72 °C for 1.5 min. The PCR product was purified with MiniBEST DNA Fragment Purification Kit (TaKaRa, Shiga, Japan) and constructed into pM19-T vector (TaKaRa, Shiga, Japan), following the manufacturer’s instruction. Then, the plasmid was transformed into Trans5α competent cells (TransGen Biotech, Beijing, China) for Sanger sequencing.

### 4.4. Sequence Analysis

The nucleotide sequence and deduced amino acid sequence of *LvCD14L* (GenBank No. ON623506) were analyzed by the BLAST algorithm (NCBI, blast.ncbi.nlm.nih.gov/Blast.cgi (accessed on 21 April 2022)) and ORF finder (https://www.ncbi.nlm.nih.gov/orffinder/ (accessed on 21 April 2022)). The LRR motif was predicted by the LRR search servers (http://lrrsearch.com/ (accessed on 21 April 2022)). The hydrophobic region was analyzed by PEPTIDE 2.0 servers (www.peptide2.com/N_peptide_hydrophobicity_hydrophilicity.php (accessed on 21 April 2022)). The signal peptide was predicted using CBS prediction servers (https://services.healthtech.dtu.dk/services/SignalP-5.0/ (accessed on 21 April 2022). Multiple sequences alignment was calculated by DNAMAN software (Version 7.0) and Sequence Manipulation Suite servers (https://www.bioinformatics.org/sms2/index.html (accessed on 21 April 2022). The sequence information of CD14s and VLRs from different species was downloaded from NCBI and is listed in Table S3. The three-dimensional (3D) structure model was calculated by SWISS-MODEL (https://swissmodel.expasy.org/ (accessed on 21 April 2022). The subcellular localization analysis was analyzed by Cell-PLoc 2.0 server (http://www.csbio.sjtu.edu.cn/bioinf/Cell-PLoc-2/ (accessed on 21 April 2022).

### 4.5. Tissue Distribution Analysis

The expression levels of *LvCD14L* in different tissues were detected by quantitative real-time PCR (qPCR) with THUNDERBIRD SYBR qPCR Mix (TOYOBO, Osaka, Japan) and primers *LvCD14L*-qF/qR. Primers 18S-F and 18S-R were also synthesized to measure the expression of the internal reference gene 18S rRNA. The program of qPCR contained one cycle of 95 °C for 1 min, followed by 40 cycles of 95 °C for 15 s, 56 °C for 15 s, 72 °C for 30 s and a melting-curve analysis added to the end of each reaction to verify the specificity of the product. The raw data of qPCR were processed with the 2^−ΔΔCT^ method [46]. The primers used in this section are listed in Table S2.

### 4.6. Pathogen Challenge and Gene Expression Analysis

Pathogenic *V. parahaemolyticus* was cultured in the tryptic soy broth (TSB) medium (LuQiao, Beijing, China) with additional 2% (g/mL) NaCl at 30 °C. The bacteria number was counted using a blood cell counting plate under a microscope and was diluted to 1 × 10^4^ CFU/μL with phosphate buffered saline (PBS) buffer. In total, 120 shrimp were equally divided into the infection group and the control group. The shrimp in the infection group were injected individually with 2 × 10^5^ CFU *V. parahaemolyticus*. The shrimp in the control group were injected with an equal volume of PBS buffer. Different tissues including hepatopancreas and hemocytes from five individuals were collected as one sample at 3 h, 6 h, 12 h and 24 h after injection. Each group contained three replicates at all time points. The total RNA of each sample was extracted and reverse transcribed into cDNA as described in section “Total RNA extraction and cDNA synthesis”. The expression levels of *LvCD14L* were detected by qPCR as described in the section “Tissue distribution analysis”.

### 4.7. DsRNA Synthesis and Knockdown of LvCD14L

The template for *LvCD14L* dsRNA synthesis was amplified with primers *LvCD14L*-dsF and *LvCD14L*-dsR (Table S2). The PCR product was purified with the Gel Extraction Kit (OMEGA, Norcross, GA, USA). DsRNA was synthesized using TranscriptAid T7 High Yield Transcription Kit (Thermo Fisher Scientific, Waltham, MA, USA) following the manufacturer’s protocol. A 289 bp dsRNA of the EGFP gene was synthesized as a negative control. The DNA template for EGFP dsRNA synthesis was amplified from the pEGFPN1 plasmid with primers EGFP-dsF and EGFP-dsR (Table S2). Thirty shrimp were separated into two groups, named the dsLvCD14L group and the dsEGFP group, to detect the optimal silencing dose. The shrimp in each group were divided into three sub-groups and injected with 3, 6 and 12 μg dsRNA per shrimp, respectively. At 48 h after dsRNA injection, the hepatopancreas and hemocytes of five individuals in each sub-group were collected and the total RNA of the samples was extracted. The expression levels of *LvCD14L* in samples were detected by RT-qPCR as described in the sections “Total RNA extraction and cDNA synthesis” and “Tissue distribution analysis”.

### 4.8. Gene Expression and Bacteria Detection in Shrimp after LvCD14L Knockdown and V. parahaemolyticus Infection

In total, 75 shrimp were equally divided into three groups including the dsLvCD14L group, the dsEGFP group and the PBS group. Each shrimp in the dsLvCD14L group and the dsEGFP group was injected with 6 μg corresponding dsRNA, and shrimp in the PBS group were injected with an equal volume of PBS buffer. At 48 h after dsRNA injection, the tissues including the hepatopancreas and hemocytes from 15 individuals were collected as three parallel samples in each group. The interference efficiency of *LvCD14L* dsRNA was verified as described in Section 4.7. The expression levels of *LvDorsal* (GenBank No. FJ998202) and *LvALF2* (GenBank No. EW713396) in hemocytes were detected by RT-qPCR as described in the sections “Total RNA extraction and cDNA synthesis” and “Tissue distribution analysis”. The primers used in this section were listed in Table S2.

The rest of shrimp were injected with 3 × 10^4^ cfu *V. parahaemolyticus* individually. At 24 h after pathogen injection (hpi), the hepatopancreases of each shrimp were collected and three individuals from the same group were mixed as one sample. Three biological replicates were prepared for each treatment. The collected hepatopancreas samples were crushed in sterile PBS buffer, and seeded onto the thiosulfate citrate bile salts sucrose (TCBS) agar culture medium (LuQiao, Beijing, China). After 16 h culture, the number of total viable bacteria and *V. parahaemolyticus* were counted, and the dominant single colonies were picked and identified by the 16s rDNA sequencing method.

### 4.9. Recombinant Expression and Purification of LvCD14L

The DNA fragment encoding the mature protein of LvCD14L was amplified with a pair of primers *LvCD14L*-rF/rR (Table S2) and was purified with a MiniBEST DNA Fragment Purification Kit (TaKaRa, Shiga, Japan). The expression vector pET32a was digested by restriction enzymes *Eco*R Ⅰ and *Bam*H Ⅰ (TaKaRa, Shiga, Japan). The purified DNA fragment was constructed into the linearized vector by an In-Fusion HD Cloning Kit (TaKaRa, Shiga, Japan), and the plasmid was transformed into *Escherichia coli* BL21-DE3 competent cells (TransGen, Beijing, China) according to the manufacturer’s instruction. The expression of rLvCD14L was induced by the addition of Isopropyl-b-d-thio-galactoside (IPTG) to a final concentration of 1 mM at 16 °C overnight. The soluble protein was purified with a HisTALON Gravity Column Purification Kit (Clontech, Mountain View, CA, USA) according to the manufacturer’s instruction. The pET32a vector protein was also expressed and purified as a control. Purity of the recombinant protein was confirmed by Sodium dodecyl sulfate-Polyacrylamide gel electrophoresis (SDS-PAGE) and visualized with eStain^TM^ L1 Protein Staining System (GenScript, Piscataway, NJ, USA). The concentration of the recombinant protein was measured with the BCA Protein Quantification Kit (Vazyme, Nanjing, China) according to the manufacturer’s protocol. The purified recombinant protein was dialyzed into PBS buffer with a dialysis bag (Solarbio, Beijing, China).

### 4.10. Microbial Cell Wall Polysaccharides Binding Assay

The pathogen-associated molecular pattern (PAMP) binding activity of rLvCD14L was detected by an enzyme-linked immunosorbent assay (ELISA). The microbial cell wall polysaccharides including LPS, PGN and dextran with a concentration of 200 μg/mL were added and coated to wells of flat-bottom microtiter plates. After incubation at 4 °C overnight, the wells were washed three times with PBST (0.05% Tween 20 in PBS). Then, the wells were blocked with 3% BSA at 37 °C for 1 h and washed three times with PBST. The rLvCD14L or rTrx (containing 1 mg/mL BSA) with a concentration gradient including 20 μg/mL, 10 μg/mL, 5 μg/mL, 2.5 μg/mL, 1.25 μg/mL, 0.625 μg/mL and 0.3125 μg/mL was incubated at room temperature for 3 h and washed three times with PBST. PBS buffer containing 1 mg/mL BSA was incubated with PAMPs as a negative control. The wells were incubated with His-Tag (27E8) Mouse mAb (Cell Signaling Technology, Danvers, MA, USA) at 37 °C for 1 h and washed three times with PBST buffer. Then, the wells were incubated with anti-mouse IgG and HRP-linked antibody (Cell Signaling Technology, Danvers, MA, USA) and washed three times with PBST buffer. The chromogenic reaction was generated using the EL-TMB Chromogenic Reagent kit (Sangon Biotech, Shanghai, China), according to the manufacturer’s instruction. Absorbance at 450 nm was detected by the precision micro-plate reader (TECAN infinite M200 PRO, Salzburg, Austria). All the experiments were performed in triplicate.

### 4.11. Minimal Inhibitory Concentration (MIC) Assay

Bacteria strains including *V. parahaemolyticus*, *V. harveyi*, *S. aureus* and *E. coli* were cultured to the logarithmic phase and were counted using a blood cell counting plate under an optical Nikon TS100 microscope (Nikon, Tokyo, Japan). The bacteria were diluted to 1 × 10^4^ cfu/mL with PBS buffer. The recombinant protein was diluted to the concentration of 1 mg/mL, 0.500 mg/mL, 0.250 mg/mL, 0.125 mg/mL, 0.0625 mg/mL and 0.0313 mg/mL using PBS buffer. Then, 50 µL of recombinant protein and 50 µL bacteria were added into each well of the 96-well plates and incubated at room temperature for 2 h. After incubation, 100 µL Tryptic Soy Broth (TSB) or Luria-Bertani (LB) medium was added and cultured at the appropriate temperature of each strain overnight. Each treatment was performed in triplicate. After cultivation, the absorbance at 600 and 560 nm for Gram-positive bacteria and Gram-negative bacteria was detected with a precision micro-plate reader (TECAN infinite M200 PRO, Salzburg, Austria), respectively.

### 4.12. Bacterial Agglutination Experiment

The bacterial agglutination experiment was carried out according to previous study with some modifications [47]. *V. parahaemolyticus* were cultured to the logarithmic phase and harvested by centrifugation at 2000× *g* for 10 min. The pellets were re-suspended with sterilized PBS at a density of 1 × 10^8^ cfu/mL after washing with PBS three times. The bacteria were labeled with 0.1 mg/mL fluorescein isothiocyanate (FITC) and slowly shaken overnight in the dark. Then, the FITC-labeled bacteria were rinsed with PBS and re-suspended in PBS at a density of 1 × 10^7^ cfu/mL and mixed with an equal volume of 1 mg/mL rLvCD14L. The PBS and rTrx were used as negative controls. Each treatment was performed in triplicate. After incubation at room temperature for 1 h, the treated cells were observed under an optical Nikon TS100 microscope (Nikon, Tokyo, Japan).

### 4.13. Cellular Localization

The DNA fragments with 15 bp terminal repeats of LvCD14L signal peptide (SP) and hydrophobic tail (HT) were amplified from pM19-T-LvCD14L. The DNA fragment with 15 bp terminal repeats of EGFP was amplified from pEGFP-N1. The purified DNA fragments were constructed into the linearized pEGFP-N1 vector with an In-Fusion HD Cloning Kit (TaKaRa, Shiga, Japan), and the plasmid was transformed into *Escherichia coli* Trans 5α competent cells (TransGen, Beijing, China) following the manufacturer’s protocol. The 293T cells were seeded into the 6-well plate (Corning, NY, USA) and Glass Bottom Cell Culture Dish (NEST, Beijing, China). The plasmids SP + EGFP, SP + EGFP + HT and EGFP-N1 were extracted with an Endo-free Plasmid Mini Kit (OMEGA, Buffalo, NY, USA) and transfected into the 293T cells using Lipofectamine 3000 (Invitrogen, Waltham, MA, USA) according to the manufacturer’s instruction. At 16 h after transfection, the cells in the Glass Bottom Cell Culture Dish were washed with PBS buffer three times and stained with Dil solution (Beyotime, Shanghai, China) for 30 min and Hoechst 33,342 solution (Beyotime, Shanghai, China) for 20 min. The cells were washed with PBS buffer three times after each stain step. Then, the cells were observed with an LSM 900 laser scanning confocal microscope (Zeiss, Oberkochen, Germany). At 24 h after transfection, the cells in 6-well plate and culture medium were collected. The cells were lysed at 4 °C for 10 min by cell lysis buffer (Beyotime, Shanghai, China). The proteins in medium were collected with saturated ammonium sulfate and resolute with PBS buffer. The samples were boiled with SDS-loading buffer for 10 min and detected by Western blot. The samples were transferred onto a polyvinylidene fluoride (PVDF) membrane by eBlot™ L1 Fast Wet Transfer System (GenScript, Piscataway, NJ, USA). The membrane was blocked with 5% skim milk which dissolved with TBS tween (TBST) buffer (TBS buffer with 0.1% tween-20) for 2 h. The membrane was incubated with Mouse anti GFP-Tag mAb (ABclonal, Wuhan, China) and GAPDH Mouse mAb (ABclonal, Wuhan, China) at 4 °C overnight and washed three times with TBST buffer. Then the membrane was incubated with anti-mouse IgG, HRP-linked antibody (ABclonal, Wuhan, China). The bands were visualized with BeyoECL Plus Kit (Beyotime, Shanghai, China) according to the manufacturer’s protocol. All the experiments were performed in triplicate.

### 4.14. Recombinant Protein Injection and Bacterial Infection

In total, 120 healthy shrimp were equally divided into an rLvCD14L group and an rTrx group. The shrimp in the two groups were injected with 10 μg corresponding recombinant protein and 1 × 10^4^ cfu *V. parahaemolyticus.* At 6 h, 12 h, 24 h and 48 h after injection, the hemocytes from five individuals were collected as one sample and each time point contained three replicates. The total RNA was extracted, and the cDNA was synthesized as described in the section “Total RNA extraction and cDNA synthesis”. The expression levels of *LvDorsal* and *LvALF2* in hemocytes were detected by qPCR as described in the section “Tissue distribution analysis”.

### 4.15. Co-Immunoprecipitation (Co-IP)

A Co-IP assay was performed to examine the interaction of LvCD14L with TLRs following the method described previously [48]. Briefly, the fragments of LvCD14L maturation protein and the extracellular region of LvToll1 (GenBank No. DQ923424.1) and LvToll2 (GenBank No. JN180637) without signal peptide were amplified from the cDNA of shrimp and constructed into the pDHsp/FLAG-His and pDHsp/V5-His vector using the In-Fusion HD Cloning Kit (TaKaRa, Shiga, Japan), respectively. All primers used in the construction of expression plasmids are listed in Table S2. The plasmids were transfected into the Sf9 cells using Lipofectamine 3000 (Invitrogen, Waltham, MA, USA) following the manufacturer’s protocol. After overnight cultivation, the cells were heat-shock treated at 42 °C for 30 min. Then, the cells were cultured at 27 °C overnight after heat-shock treatment. The cells were lysed at 4 °C for 10 min with cell lysis buffer (Beyotime, Shanghai, China). The lysate was centrifuged at 12,000 rpm for 10 min, and the supernatant was collected and incubated with Anti-FLAG M2 Magnetic beads (Sigma, Ronkonkoma, NY, USA) at 4 °C for 2 h. Before incubation, part of the supernatant was preserved as Input. After incubation, the magnetic beads and proteins were collected with a Magnetic Separation Device (Sangon Biotech, Shanghai, China) and then washed with PBS three times. After the last wash, the magnetic beads were re-suspended and boiled with SDS-loading buffer for 10 min. The supernatant was collected as CoIP sample. All samples were detected by Western blot with V5-Tag (D3H8Q) Rabbit mAb and DYKDDDDK Tag (D6W5B) Rabbit mAb (Cell Signaling Technology, Danvers, MA, USA) as described in the section “Cellular Localization”. All the experiments were performed in triplicate.

## Data Availability

The original contributions presented in the study are included in the article/Supplementary Material. Further inquiries can be directed to the corresponding authors.

## Supplementary Materials

The supporting information can be downloaded at: https://www.mdpi.com/article/10.3390/ijms24097770/s1.
