# Supplementary material for: LvCD14L Acts as a Novel Pattern Recognition Receptor and a Regulator of the Toll Signaling Pathway in Shrimp
Source: Int J Mol Sci. 2023 Apr 24;24(9):7770. doi: 10.3390/ijms24097770 (PMC10178686; doi:10.3390/ijms24097770)
Supplement: Supplementary file 1 [file ijms-24-07770-s001.zip › Supporting information.pdf]

Supporting information

FIGURE S1. The agglutination activity of rLvCD14L to *V. parahaemolyticus*. rTrx and PBS was used as negative control.

Table S1. Nucleotide sequences of primers used in the present study.

Table S2. Sequence information of genes from different species in multiple sequence alignment.

Table S3. Minimal inhibitory concentration of rLvCD14L on different bacteria.
