# Supplementary figures and images for: LvCD14L Acts as a Novel Pattern Recognition Receptor and a Regulator of the Toll Signaling Pathway in Shrimp
Source: Int J Mol Sci. 2023 Apr 24;24(9):7770. doi: 10.3390/ijms24097770 (PMC10178686; doi:10.3390/ijms24097770)

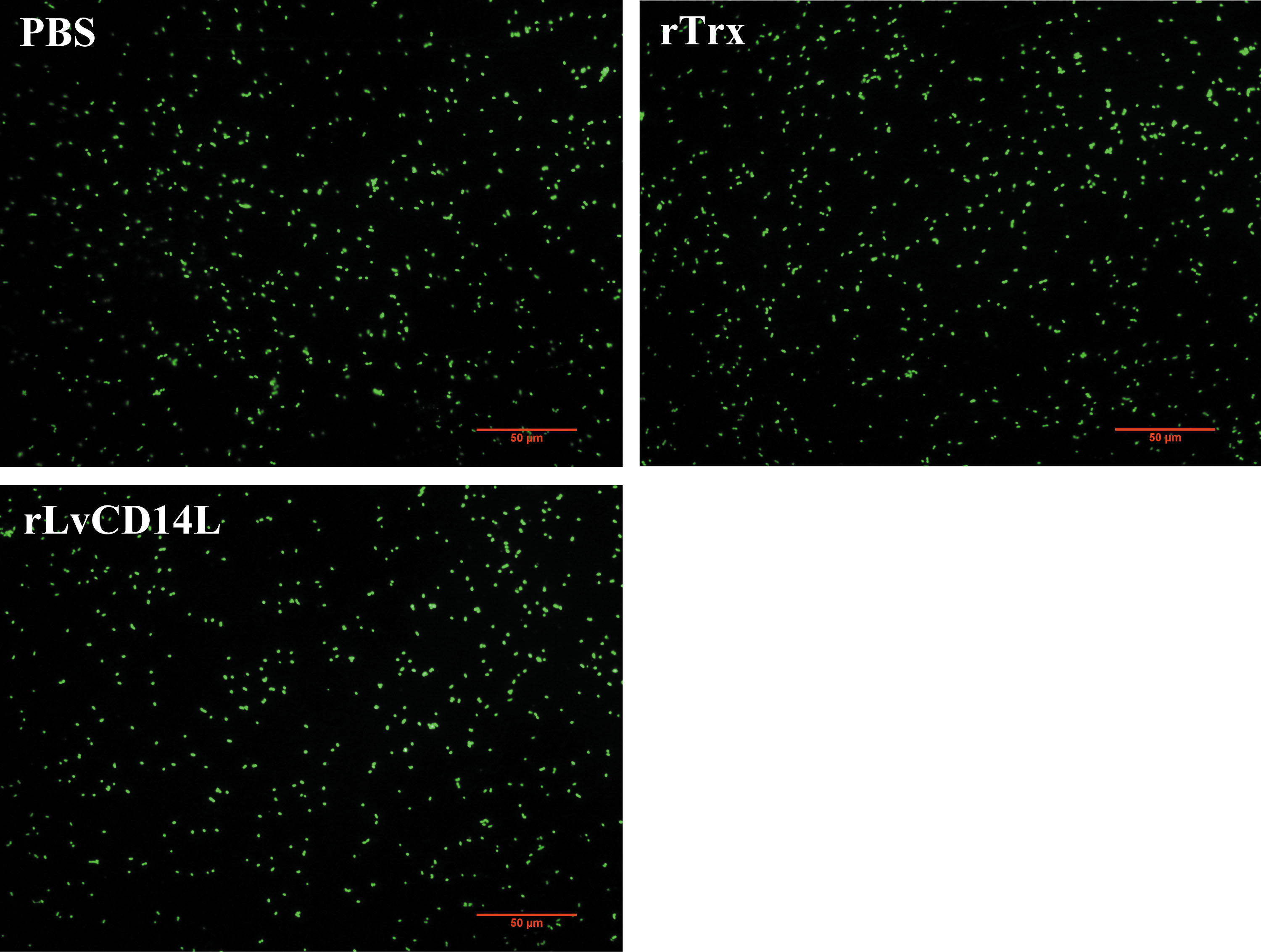

Supplement: Supplementary file 1 [file ijms-24-07770-s001.zip › Figure S1.jpg]
